# Is It Safe to Switch from a Standard Anterior to Retzius-Sparing Approach in Robot-Assisted Radical Prostatectomy?

**DOI:** 10.3390/curroncol30030261

**Published:** 2023-03-17

**Authors:** Edward Lambert, Charlotte Allaeys, Camille Berquin, Pieter De Visschere, Sofie Verbeke, Ben Vanneste, Valerie Fonteyne, Charles Van Praet, Nicolaas Lumen

**Affiliations:** 1 ERN eUROGEN Accredited Centre, Department of Urology, Ghent University Hospital, 9000 Ghent, Belgiumcharles.vanpraet@uzgent.be (C.V.P.);; 2 Junior ERUS/YAU Working Group on Robot-Assisted Surgery of the European Association of Urology, 6803 AA Arnhem, The Netherlands; 3 Department of Radiology and Nuclear Medicine, Ghent University Hospital, 9000 Ghent, Belgium; 4 Department of Pathology, Ghent University Hospital, 9000 Ghent, Belgium; 5 Department of Radiation Oncology, Ghent University Hospital, 9000 Ghent, Belgium; 6 Department of Radiation Oncology (MAASTRO), GROW—School for Oncology and Developmental Biology, Maastricht UMC, 6229 HX Maastricht, The Netherlands

**Keywords:** radical prostatectomy, Retzius-sparing, urinary continence, erectile dysfunction, biochemical recurrence, robotic surgery

## Abstract

Background: Retzius-sparing robot-assisted radical prostatectomy (RS-RARP) has been shown to lead to better outcomes regarding early continence compared to standard anterior RARP (SA-RARP). The goal of this study was to assess the feasibility and safety of implementing RS-RARP in a tertiary center with experience in SA-RARP. Methods: From February 2020, all newly diagnosed non-metastatic prostate cancer patients for whom RARP was indicated were evaluated for RS-RARP. Data from the first 100 RS-RARP patients were prospectively collected and compared with data from the last 100 SA-RARP patients. Patients were evaluated for Clavien Dindo grade ≥3a complications, urinary continence after 2 and 6 weeks, 3, 6 and 12 months, erectile function, positive surgical margins (PSMs) and biochemical recurrence (BCR). Results: There was no significant difference in postoperative complications at Clavien-Dindo grade ≥3a (SA-RARP: 6, RS-RARP: 4; *p* = 0.292). At all time points, significantly higher proportions of RS-RARP patients were continent (*p* < 0.001). No significant differences in postoperative potency were observed (52% vs. 59%, respectively, *p* = 0.608). PSMs were more frequent in the RS-RARP group (43% vs. 29%, *p* = 0.034), especially in locally advanced tumors (pT3: 64.6% vs. 43.8%, *p* = 0.041—pT2: 23.5% vs. 15.4%, *p* = 0.329). The one-year BCR-free survival was 82.6% vs. 81.6% in the SA-RARP and RS-RARP groups, respectively (*p* = 0.567). The median follow-up was 22 [18–27] vs. 24.5 [17–35] months in the RS-RARP and SA-RARP groups, respectively (*p* = 0.008). Conclusions: The transition from SA-RARP to RS-RARP can be safely performed by surgeons proficient in SA-RARP. Continence results after RS-RARP were significantly better at any time point. A higher proportion of PSMs was observed, although it did not result in a worse BCR-free survival.

## 1. Introduction

Prostate cancer is the second most prevalent cancer worldwide, with one in nine men being diagnosed with the disease during their life. With an estimated 1.4 million diagnoses and 375,000 deaths in 2020, prostate cancer is the second most commonly diagnosed and fifth most lethal cancer in men [1].

Radical prostatectomy is, besides external beam radiation therapy and brachytherapy, a recommended treatment option for localized and selected cases of locally advanced prostate cancer. Different surgical approaches have been reported, but all surgical techniques—including open retropubic prostatectomy, laparoscopic prostatectomy or robot-assisted radical prostatectomy—have a risk of temporary or persistent urinary incontinence [2]. Urinary incontinence after radical prostatectomy may strongly affect the quality of life of prostate cancer patients [2].

In the last decade, robot-assisted radical prostatectomy has gained popularity due to its minimally invasive character and improved dexterity, with a decrease in blood loss, postoperative pain, and duration of hospitalization [3]. In the standard anterior robot-assisted radical prostatectomy (SA-RARP), the Retzius space is opened in a similar fashion to the open retropubic and laparoscopic approach [4]. 

In 2010 Galfano, et al. [5] published an alternative surgical approach for robot-assisted radical prostatectomy through the Douglas pouch in which the Retzius space remains intact (Retzius-sparing robot-assisted radical prostatectomy, RS-RARP). This innovative surgical technique avoids damage to the supportive fascial structures of the bladder. In comparison to SA-RARP, the Santorini plexus, the endopelvic fascia, puboprostatic ligaments, and the detrusor apron remain intact in RS-RARP [6]. Different prospective trials have reported better outcomes in terms of early continence in RS-RARP than SA-RARP, with immediate continence results ranging from 51–76% for RS-RARP compared to 21–60% in SA-RARP [7,8,9,10]. However, higher proportions of positive surgical margins have been reported with uncertain biochemical recurrence-free survival results [10].

Therefore, the goal of this study was to assess the feasibility and safety of the implementation of RS-RARP in a medium volume (ca. 100 cases/year) tertiary center with experience in SA-RARP, with a special focus on functional and oncological outcomes.

## 2. Materials and Methods

RS-RARP was implemented on 7 February 2020 in our institution. All patients with newly diagnosed prostate cancer for whom radical prostatectomy was indicated were evaluated for RS-RARP. The decision to perform RS-RARP was of the surgeon’s choice, and patients with anterior tumors close to the bladder neck continued to be treated with ‘classic’ SA-RARP. Our RS-RARP procedure has previously been described in detail [11]. In brief, the RS-RARP procedure starts with the incision of the peritoneum above the vasa deferentes up until the Douglas pouch, where the peritoneal fold is incised. The vasa deferentes are dissected and cut at the tip of the seminal vesicles. Denonvilliers’ fascia is peeled off of the seminal vesicles, and they are dissected completely. The lateral side of the prostate is dissected by retracting the seminal vesicles medially. To facilitate access to the prostate and bladder neck, stay sutures are placed on the upper part of the peritoneal incision by use of a straight needle which is placed through the abdominal wall.

For a nerve-sparing approach, a plane is created between Denonvilliers’ fascia and the prostatic fascia up to the apex of the prostate. The neurovascular bundle is peeled off of the prostatic fascia laterally by blunt dissection. The prostatic pedicle is then clipped and transected. The plane is followed anteriorly, and the bladder is peeled from the prostate. Bladder neck dissection starts at its posterior side, just anteriorly of the seminal vesicles and the bladder neck is isolated. After bladder neck transection, two marking sutures are placed at the 6 and 12 o’clock positions to evert the mucosa and aid in vesicourethral anastomosis. The anterior surface of the prostate is dissected, sparing Santorini’s plexus, the puboprostatic/pubovesical ligaments and the Retzius space. The dissection continues apically with transection of the urethra. A vesicourethral anastomosis is performed with two barbed sutures, starting at the 12 o’clock position on the bladder neck and ending at the 6 o’clock position on the urethra after complete and watertight approximation of the bladder neck and urethra. After a leak test is performed, the peritoneal incision is closed using a barbed suture [11]. SA-RARP was performed as described by Menon, et al. and Bianchi et al. [4,12]. All surgical interventions were performed by two surgeons: N.L. is an experienced robotic surgeon who previously performed about 800 cases of SA-RARP, while C.V.P. was a novice robotic surgeon at the time of this study who had previously performed about 30 independent SA-RARP cases. The RS-RARP was started by N.L. after analyzing the surgical technique from literature and online surgical videos. After 10 cases successfully performed by N.L., C.V.P. started with the technique, was mentored by N.L. and performed them independently after 10 performed cases.

Data from the first 100 RS-RARP patients were prospectively collected and compared with the data from the last 100 SA-RARP patients before February 2020 (period: August 2018–January 2020). Data from the SA-RARP patients were retrospectively analyzed. No performed RS-RARP case was excluded. In our center, the postoperative policy after both RS-RARP and SA-RARP follows a standardized protocol in which the patient remains hospitalized until the time of bladder catheter removal. The urinary catheter is removed on postoperative day three without cystography in case of a negative intraoperative leakage test. When the intraoperative leakage test is positive, a cystography is performed on postoperative day three. The urinary catheter is only removed when this cystography shows a watertight vesicourethral anastomosis. In the case of normal micturition after catheter removal, the patient can leave the hospital on postoperative day three. Both treatment groups were subjected to the same follow-up protocol with control visits at 2 weeks, 6 weeks, 3, 6, and 12 months postoperatively, at which the oncological and functional results were evaluated, and possible complications were reported. All RS-RARP patients had a minimum of 12 months follow-up.

Demographic, pre- and perioperative data were collected and compared between both groups. Complications within 90 days after surgery were reported using the standardized Clavien-Dindo classification for surgical complications [13]. Postoperative continence status was categorized as continent (dry or loss of only some drops per day with the use of maximal 1 safety pad) vs. incontinent [14]. Postoperative erectile function was assessed by questioning the patient at each follow-up visit and was categorized as potent (defined as the ability to achieve and sustain an erection firm enough for satisfactory sexual performance through penetration with or without pharmacological assistance) vs. impotent.

The endpoints of this study were Clavien-Dindo grade 3a or higher complications, urinary continence after 2 and 6 weeks and 3, 6 and 12 months, postoperative erectile function in preoperative potent men, positive surgical margin rate, PSA after 3 months and 1-year biochemical recurrence-free survival. Biochemical recurrence was defined as PSA >0.20 ng/mL or treatment with salvage radiotherapy. Salvage radiotherapy was performed when three consecutive PSA rises occurred, even when PSA levels did not exceed 0.2 ng/mL. A positive surgical margin was defined as a tumor extending into the inked surface of the prostatectomy specimen at microscopic pathological examination.

Continuous variables were reported as the median and interquartile range (IQR) and compared between both groups using the Mann–Whitney U test. Categorical variables were compared using the Chi-square or Fisher’s exact test whenever applicable. A *p*-value < 0.05 was considered statistically significant. Urinary continence rates and biochemical recurrence-free survival were calculated using Kaplan–Meier statistics and compared between the two groups using the log-rank test. All statistical analyses were performed using SPSS v28 statistical software (IBM SPSS, Chicago, IL, USA). The study was conducted according to the guidelines of the Declaration of Helsinki and approved by the Institutional Review Board of the Ghent University Hospital (EC UZG 2019/1506).

## 3. Results

A comparison of demographic and preoperative parameters between the SA-RARP and RS-RARP groups is summarized in Table 1.

In the RS-RARP group, the total operative time was longer compared to the SA-RARP group (160 min vs. 147.5 min, *p* = 0.005) (Table 2). Positive intraoperative leakage tests were significantly more frequent in the RS-RARP group (17% vs. 7 %, *p* = 0.028). The incidence of positive intraoperative leakage tests was significantly higher in cases 1–33 compared to cases 67–99 (9/33 [27%] vs. 2/33 [6%], *p* = 0.044) of RS-RARP. There were no significant differences in estimated blood loss, duration of urinary catheterization or length of hospital stay between the RS-RARP and SA-RARP groups (Table 2).

One case of RS-RARP was aborted due to the intraoperative finding of extreme perivesical fibrosis after previous mitomycin C leakage. The abortion of surgery was, therefore, not related to the surgical technique of RS-RARP. No case was converted from RS-RARP to SA-RARP.

Postoperative complications are summarized in Table 3. Overall, significantly fewer complications occurred in the RS-RARP group (*p* = 0.028). However, there was no significant difference in postoperative complications Clavien-Dindo grade ≥3A between the SA-RARP and RS-RARP groups (6% vs. 2%, respectively, *p* = 0.292).

**Figure 1:**
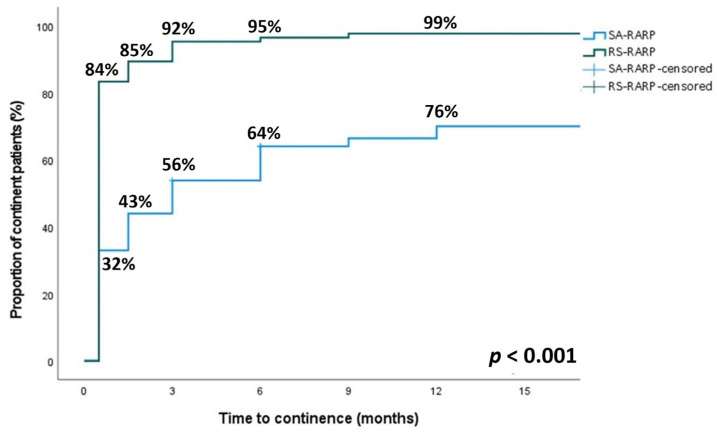
Comparison of the relative continence rate at 2 weeks, 6 weeks, 3 months, 6 months and 12 months after surgery after RS-RARP (green line) and SA-RARP (blue line). A significant difference in continence rate was observed between both groups at any time point after surgery (*p* < 0.001).

At all time points during postoperative follow-up, a significantly higher proportion of patients in the RS-RARP group was continent (*p* < 0.001) (Figure 1). The immediate urinary continence at two weeks postoperatively and continence at one year postoperatively were significantly higher in the RS-RARP group (84% vs. 32% [*p* < 0.001] and 99% vs. 76% [*p* < 0.001], respectively).

Functional and oncological outcomes are summarized in Table 4. A significantly higher proportion of patients in the RS-RARP group were potent preoperatively (RS-RARP 63/100 vs. SA-RARP 48/100, *p* < 0.001). Of all patients who were potent preoperatively, 25/48 (52%) in the SA-RARP group and 37/63 (59%) in the RS-RARP group (*p* = 0.608) regained potency during postoperative follow-up (Table 4).

**Figure 2:**
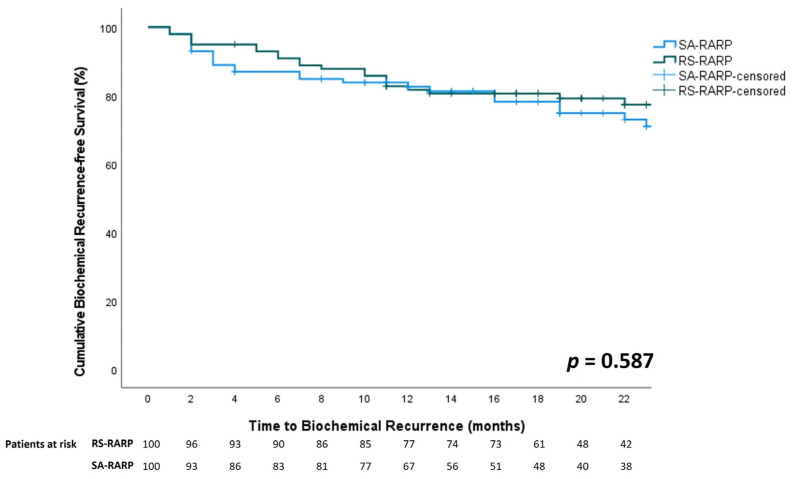
Comparison of biochemical recurrence free-survival between RS-RARP (green line) and SA-RARP (blue line).

Overall, a significantly higher proportion of positive surgical margins was observed in the RS-RARP group compared to the SA-RARP group (43% vs. 29%, *p* = 0.034). When stratified for pathological T-stage, a significantly higher proportion of positive surgical margins was observed in the RS-RARP group in locally advanced prostate cancer (64.6% vs. 43.8%, *p* = 0.041), but not in localized prostate cancer (pT2: 23.5% vs. 15.4%, *p* = 0.329). In both groups, 85/100 (85%) patients had an undetectable PSA at 3 months postoperatively (*p* = 1). Biochemical recurrence was observed in 27/100 (27%) of SA-RARP patients vs. 23/100 (23%) of RS-RARP patients (*p* = 0.540). Median time to biochemical recurrence was 17 (11–29) months vs. 19 (14–26) months (*p* = 0.394) in the SA- and RS-RARP groups, respectively. The one-year biochemical recurrence-free survival rate was 82.6% vs. 81.6% in the SA-RARP and RS-RARP groups, respectively (*p* = 0.587, Figure 2). Salvage radiotherapy was performed in 21/100 (21%) patients in both groups (*p* = 0.605). Of all patients who underwent salvage radiotherapy, 4/21 (19%) patients in the RS-RARP group and 8/21 (38%) patients in the SA-RARP group had a combination of ISUP 4-5 + pT3 + R1 disease (*p* = 0.17). Median follow-up in the SA-RARP and RS-RARP groups was 24.5 [17,18,19,20,21,22,23,24,25,26,27,28,29,30,31,32,33,34,35] vs. 22 [18,19,20,21,22,23,24,25,26,27] months, respectively (*p* = 0.008).

To evaluate predictors for biochemical recurrence after RS-RARP, a univariate and multivariate logistic regression analysis was performed in the RS-RARP group (Table 5). Significant covariates in univariate regression were selected for multivariate logistic regression. Pathological T-stage proved to be an independent predictor for biochemical recurrence after RS-RARP (OR 14.99 [2.77–81.11], *p* = 0.002).

## 4. Discussion

The goal of this study was to evaluate the safety of switching from SA-RARP to RS-RARP by surgeons standardly performing SA-RARP. The results of this study show that shifting from a standard anterior to a Retzius-sparing approach is indeed safe, without a significant increase in high-grade complications or bleeding. The significantly longer operative time and the higher proportion of positive intraoperative leakage tests in RS-RARP that were encountered in this study can be attributed to the technical learning curve a surgeon has to pass through, as was previously reported by Galfano et al. [15]. However, since the duration of catheter stay and hospital stay were equal in both the RS-RARP and SA-RARP groups in our study, the observed statistical differences in operative time and positive leakage tests do not seem clinically relevant.

At every postoperative time point, continence was significantly better in the RS-RARP group compared to the SA-RARP group. The two-week continence rate in the RS-RARP group (84%) is higher compared to previously published data, in which early continence rates of 51–76% have been reported [7,9,16,17,18]. Since our definition of continence was similar to other published data [9,15,19,20,21,22], this could be due to small differences in surgical approach and techniques, which we described in detail previously [11].

The one-year postoperative continence rate in our study was similar to previously reported data, in which one-year continence rates after RS-RARP of 90–100% have been reported [17,19,20,21,23,24]. The one-year continence rate after SA-RARP in our study is at the lower bound of those reported in literature, which greatly vary between 70–95% [25,26]. When comparing the continence rate between the RS-RARP and SA-RARP at one year postoperatively, a significantly higher continence rate remained in the RS-RARP group in our study. The previously published literature is contradictory regarding this topic. Meta-analyses of Rosenberg et al. [10], Phukan et al. [27], and Barakat et al. [14] did not show a difference in one-year continence rates between RS-RARP and SA-RARP populations, whereas meta-analyses of Checcucci, et al. [28] and Tai, et al. [29] did find a significant difference in one-year continence rates between both groups. The significant difference in continence rate between the RS-RARP and SA-RARP groups of this study may have been influenced by uncontrolled confounders, as 8% of SA-RARP patients underwent previous TURP, whereas none of the RS-RARP patients underwent TURP before.

After SA-RARP, the one-year continence rate varies substantially and is dependent on several factors, including the surgeon’s experience and annual caseload [25]. The one-year continence rate of RS-RARP, on the other hand, is rather similar between series [17,19,20,21,23,24]. This may explain the contradicting results of several meta-analyses comparing SA-RARP to RS-RARP. Although both our surgeons were at the beginning of their learning curve for RS-RARP, patients treated with RS-RARP already had good short- and long-term continence, demonstrating that continence outcome may be less dependent on the surgeon’s experience in RS-RARP compared to SA-RARP.

We observed no significant difference in erectile function recovery between SA-RARP and RS-RARP patients. Of the preoperatively potent patients, 59% of the RS-RARP group recovered its sexual function during follow-up. This is in line with previously published results of Galfano et al. [15] and Egan et al. [21], who reported, respectively, 52% and 65.7% of potent preoperative patients regained their sexual function during follow-up. A meta-analysis by Barakat et al. [14] also did not find a significant difference in erectile function recovery between RS-RARP and SA-RARP.

A higher proportion of positive surgical margins was observed in the RS-RARP group in this study. The overall positive surgical margin rate of 43% in our study was higher compared to previously reported positive surgical margin rates of RS-RARP of 25–30.6% [15,19,30]. This may be related to the high number of 48% pT3 tumors in this series, whereas Dalela et al. [19] (who reported a positive surgical margin rate of 25% in RS-RARP) only reported on patients with low- or intermediate-risk prostate cancer. Our high number of positive surgical margins in pT3 tumors of 64.6% is comparable to the positive surgical margin rate of 67.5% in pT3b tumors of RS-RARP patients reported by Abdel Raheem et al. [30]. Furthermore, this positive surgical margin rate is comparable to a previously published series of 1384 consecutive patients who underwent SA-RARP, in which positive surgical margins of 60.5% were encountered in patients with pT3-4 [31].

Although a higher proportion of positive surgical margins occurred in the RS-RARP group, no significant difference in biochemical recurrence was observed in comparison to the SA-RARP group. The one-year biochemical recurrence-free survival was similar in both groups, and the number of patients that needed salvage therapy was equal in both groups.

The one-year biochemical recurrence-free survival in the RS-RARP group of our study (81.6%) is lower than the previously reported one-year biochemical recurrence-free survival of 92.1% after RS-RARP [30], and the overall biochemical recurrence rate in the RS-RARP group in this study (23%) is higher than the previously reported overall biochemical recurrence rate of 14.8% after RS-RARP [30]. This may largely be explained by the high number of locally advanced and high-risk tumors included in this study. Galfano et al. reported similar biochemical recurrence results of 27.5% at an equal median follow-up of 22 months in a high-risk prostate cancer patient cohort undergoing RS-RARP [32]. Furthermore, biochemical recurrence in this study was more strictly defined than in other studies, in which biochemical recurrence was solely defined as PSA >0.20 ng/mL.

The significantly higher proportion of positive surgical margins in the RS-RARP group may be influenced by different factors. First and foremost, the learning curve of the surgeon has a crucial impact. Galfano, et al. reported that even after 50 cases the number of surgical margins does not significantly decrease [15]. Secondly, we hypothesize that a proportion of the positive surgical margins, as described in the pathology reports, may have been false positive due to tears in the prostatic capsule caused by intraoperative traction. In such cases, a ‘positive’ surgical margin would not have led to tumor tissue remaining in the surgical field, thus not resulting in biochemical recurrence. With RS-RARP, the surgeon remains closer to the prostate, and dissection is more blunt than sharp as compared to SA-RARP, especially at the bladder neck and anterior prostate. This hypothesis is supported by the fact that the higher number of positive surgical margins is not correlated with a higher number of patients with a biochemical recurrence, although follow-up is sufficiently long to establish biochemical recurrence. Furthermore, surgical margin status proved not to be an independent predictor for biochemical recurrence in the RS-RARP group.

Different randomized controlled trials reported on a head-to-head comparison of the SA-RARP and RS-RARP techniques. Asimakopoulos et al. [7] reported immediate continence results of 21% and 51% after SA-RARP and RS-RARP, respectively (*p* = 0.001). The recovery of urinary continence after RS-RARP was significantly faster with only 1 day compared to 21 days for SA-RARP (*p* = 0.02). Overall positive surgical margins were reported in 28.2% of RS-RARP cases, which was significantly higher than the 10.0% positive surgical margins in the SA-RARP group. Biochemical recurrence was not assessed by Asimakopoulos et al. Dalela et al. [19] reported significantly higher continence results for the RS-RARP technique, with 71% of patients being continent 1 week after catheter removal compared to 48 in the SA-RARP group (*p* = 0.01). The median time to continence was 2 and 8 days after catheter removal in the RS-RARP and SA-RARP groups, respectively (*p* = 0.02). Overall, positive surgical margins occurred in 25% and 13% of the RS-RARP and SA-RARP groups, respectively. However, only low and intermediate-risk prostate cancer patients were included in this study. Qiu et al. [17] also reported significantly higher immediate continence results of 69.1% after RS-RARP compared to 30.9% after SA-RARP. No significant differences in PSMs (14.5% vs. 23.6% in SA-RARP and RS-RARP, respectively) or BCR-FS were observed in this study.

Apart from these RCTs, different prospective and retrospective observational studies compared the efficacy and safety of RS-RARP and SA-RARP cohorts. Umari et al. [8] prospectively compared 201 SA-RARP to 282 RS-RARP patients. Immediate urinary continence was higher in the RS-RARP group (70.4% vs. 58.1%, *p* = 0.02). PSMs were reported in 15.6% and 13.9% of RS-RARP and SA-RARP, respectively (*p* = 0.600), with 33.7% and 20.3% of PSMs in pT3 patients, respectively (*p* = 0.254).

Lim et al. [18] compared data of 50 RS-RARP patients with 50 SA-RARP patients after propensity-score matching. They reported an overall PSM rate of 14% in both RS-RARP and SA-RARP patients (*p* = 1.00) and a PSM rate in pT3 disease of 41.7% and 22.2% for RS-RARP and SA-RARP, respectively (*p* = 0.64). At 4 weeks postoperatively, 70% of RS-RARP patients and 50% of SA-RARP patients were continent (*p* = 0.04). Lee et al. [33] compared data from 609 RS-RARP patients with 609 SA-RARP patients after propensity-score matching. No significant differences in complications or PSMs were observed between both groups. The continence rates at 1 month postoperatively were 45% and 9.0% in the RS-RARP and SA-RARP groups, respectively, which rose to 98% and 77% by month six postoperatively (*p* < 0.001).

Egan et al. [21] retrospectively evaluated the results of 140 consecutive RARPs (70 SA-RARP + 70 RS-RARP). No significant difference in complications was observed. At 12 months postoperatively, 97.6% and 81.4% of patients in the RS-RARP and SA-RARP groups, respectively, were continent (*p* = 0.002). The time to continence recovery was significantly faster in the RS-RARP group (44 vs. 131 days, *p* < 0.001). Positive surgical margins were observed in 34.3% and 30.0% of RS-RARP and SA-RARP patients, respectively (*p* = 0.590), with BCR occurring in 12.9% and 18.6% of patients in the RS-RARP and SA-RARP groups, respectively (*p* = 0.357). However, the median follow-up of RS-RARP patients was significantly shorter, reaching only 12.3 months.

Anil et al. [34] reported on an analysis of data from a cohort of 50 RS-RARP and 50 SA-RARP patients and retrospectively assessed the safety of the switch from an anterior to a posterior approach. They did not report a significant difference in erectile function recovery, early urinary continence recovery, continence recovery at 1 year postoperatively or positive surgical margins. Biochemical recurrence was observed in 14% and 12% of RS-RARP and SA-RARP patients, respectively (*p* = 0.766). Remarkably, no cT3 tumors were included in this study.

This study has several limitations. Firstly, this is a non-randomized study that is prone to bias. Patients with tumors close to the bladder neck or in the anterior part of the prostate were actively withheld from inclusion in the RS-RARP group. This selection bias may compromise the generalizability of the results of this study. Furthermore, the retrospective analysis of the SA-RARP data is inextricably linked to a certain level of bias in which missing data may have affected data analysis. Secondly, the Gleason score at the positive surgical margin and the extent of positive surgical margins were not reported as they are unknown. However, this information is highly relevant as some positive surgical margins may harbor a large burden of aggressive disease, while others hold a low volume of low-grade residual tumors. Since a lower Gleason score at the positive surgical margin is independently associated with a shorter margin length and a decreased risk of early biochemical recurrence [35], this information could have influenced the interpretation of the results of this study. Thirdly, the follow-up of the RS-RARP group is shorter than the follow-up of the SA-RARP group. This could have influenced the incidence of biochemical recurrence and, with that, the need for salvage radiotherapy and the possible impact of such therapies on functional outcomes. Nevertheless, we are convinced that the follow-up is sufficiently long to allow a reliable interpretation of the oncological and functional outcomes and, thus, a reliable comparison of both groups. Thirdly, erectile function was evaluated by questioning patients at each follow-up visit. However, validated questionnaires were not used to assess erectile function, which may have influenced the erectile function outcome assessment. Finally, this is a single institution evaluation from a tertiary center with a relatively small sample size, which may influence the generalizability of the results.

## 5. Conclusions

The transition from SA-RARP to RS-RARP can be performed safely by surgeons proficient in SA-RARP without a significant increase in complications, intraoperative blood loss, catheter duration, or hospital stay. In our hands, functional results of RS-RARP are significantly better with higher proportions of continent patients at any time point after surgery. Oncologically, a higher proportion of positive surgical margins was observed, although this did not lead to worse oncological outcomes at 22 months follow-up. Further follow-up is necessary to validate these results. Patients eligible for RS-RARP should be informed about the higher risk of positive surgical margins.

## Figures and Tables

**Table 1:** Patient and tumor characteristics in the SA-RARP and RS-RARP groups (reported as *N*(%) and median (interquartile range)).

		SA-RARP (*N* = 100)	RS-RARP (*N* = 100)	*p*
Age (years)		67 (62–73)	66 (61–70)	0.166
BMI (kg/m²)		26.3 (24.1–28.5)	26.3 (24.3–29.0)	0.713
ASA-score		2 (2–2)	2 (2–2)	0.766
Prostate volume (mL)		45 (34–56)	44 (34–65)	0.756
PSA (ng/mL)		8.62 (6.16–10.79)	7.38 (5.37–10.60)	0.09
Previous prostate surgery				0.009
	None	91 (91%)	100 (100%)	
	TURP	8 (8%)	0 (0%)	
	Other	1 (1%)	0 (0%)	
Biopsy ISUP Grade Group				0.176
	1	18 (18%)	15 (15%)	
	2	35 (35%)	34 (34%)	
	3	19 (19%)	32 (32%)	
	4	15 (15%)	13 (13%)	
	5	13 (13%)	6 (6%)	
cT				0.005
	T1c	26 (26%)	39 (39%)	
	T2	30 (30%)	29 (29%)	
	T3	3 (3%)	12 (12%)	
	T4	1 (1%)	0 (0%)	
	Missing	39 (39%)	18 (18%)	
cN				0.32
	N0	94 (94%)	98 (98%)	
	N1	3 (3%)	2 (2%)	
	Nx	2 (2%)	0 (0%)	
	Missing	1 (1%)	0 (0%)	
cM				0.059
	M0	66 (66%)	78 (78%)	
	Mx	34 (34%)	22 (22%)	
MRI				0.063
	PIRADS 1–2	4 (4%)	9 (9%)	
	PIRADS 3	12 (12%)	13 (13%)	
	PIRADS 4–5	78 (78%)	77 (77%)	
	Not performed/Missing	6 (6%)	1 (1%)	
iT				0.155
	T2	70 (70%)	63 (63%)	
	T3	18 (18%)	26 (26%)	
	No tumor	4 (4%)	10 (10%)	
	Not performed/missing	8 (8%)	1 (1%)	
Index Lesion Location				<0.001
	Peripheral zone	63 (63%)	83 (83%)	
	Anterior zone	20 (20%)	4 (4%)	
	Multifocal (anterior and posterior)	5 (5%)	2 (2%)	
	No MRI or no index lesion	12 (12%)	11 (11%)	

ASA: American Society of Anaesthesiologists; BMI: Body Mass Index; ISUP: International Society of Urological Pathology; MRI: Magnetic Resonance Imaging; PSA: Prostate Specific Antigen; RS-RARP: Retzius-Sparing Robot-Assisted Radical Prostatectomy; SA-RARP: Standard Anterior Robot-Assisted Radical Prostatectomy.

**Table 2:** Intraoperative and early postoperative data in the SA-RARP and RS-RARP groups.

		SA-RARP (*N* = 100)	RS-RARP (*N* = 100)	*p*
Surgeon				<0.001
	NL	96 (96%)	71 (71%)	
	CVP	4 (4%)	28 (28%)	
	Missing	0 (0%)	1 (1%)	
Operative time (mins)		147.5 (125–175)	160 (145–180)	0.005
Estimated blood loss (mL)		400 (150–400)	225 (100–300)	0.096
Positive lymph nodes		0 (0–0)	0 (0–0)	<0.001
Nerve-sparing (%)				0.005
	None	9 (9%)	6 (6%)	
	Unilateral	22 (22%)	43 (43%)	
	Bilateral	69 (69%)	50 (50%)	
	Missing	0 (0%)	1 (1%)	
Intraoperative leak test				0.028
	No leakage	93 (93%)	82 (82%)	
	Leakage	7 (7%)	17 (17%)	
	Missing	0 (0%)	1 (1%)	
Duration of urinary catheter stay (days)		3 (3–3)	3 (3–3)	0.382
Length of stay (days)		3 (3–3)	3 (3–3)	0.268

RS-RARP: Retzius-Sparing Robot-Assisted Radical Prostatectomy; SA-RARP: Standard Anterior Robot-Assisted Radical Prostatectomy.

**Table 3:** Postoperative complications according to Clavien-Dindo in the SA-RARP and RS-RARP groups.

Clavien-Dindo	SA-RARP (*N* = 100)		RS-RARP (*N* = 100)		*p*	
**0**	**79**		**88**		**0.028**	
**1**	12	Urinary retention (11) Abdominal wall hematoma (1)	9	Urinary retention (7) Prolonged suprapubic pain (1) Prolonged hematuria (1)		
**2**	9	Wound infection (1) Urinary tract infection (7) Anastomotic leakage (1)	1	Infected lymfocele (1)		
**3A**	0		1	Infected lymfocele (1)		**0.292**
**3B**	4	Cholecystitis (1) Hemoperitoneum (1) Anastomotic leakage (1) Obstructive pyelonephritis (1)	1	Gastrointestinal obstruction (1)		
**4A**	1	Hypovolemic shock (1)	0			
**4B**	1	Urosepsis (1)	0			

RS-RARP: Retzius-Sparing Robot-Assisted Radical Prostatectomy; SA-RARP: Standard Anterior Robot-Assisted Radical Prostatectomy.

**Table 4:** Functional and oncological outcomes in the SA-RARP and RS-RARP groups.

			SA-RARP (*N* = 100)	RS-RARP (*N* = 100)	*p*
Preoperative potency (*N* = 200)					<0.001
	Impotent		31 (31%)	27 (27%)	
	Potent		48 (48%)	63 (63%)	
	Missing		21 (21%)	9 (9%)	
Postoperative potency in preoperatively potent men (*N* = 111)					0.608
	Impotent		21 (44%)	25 (40%)	
	Potent		25 (52%)	37 (59%)	
	Missing		2 (4%)	1 (1%)	
Pathology T stage					0.38
	T2		52 (52%)	51 (51%)	
	T3a		32 (32%)	38 (38%)	
	T3b		16 (16%)	10 (10%)	
	Missing		0 (0%)	1 (1%)	
Pathology *N* stage					<0.001
	pN0		49 (49%)	34 (34%)	
	pN1		10 (10%)	0 (0%)	
	pNx		41 (41%)	65 (65%)	
	Missing		0 (0%)	1 (1%)	
Pathology ISUP Grade Group					0.344
	1		5 (5%)	5 (5%)	
	2		43 (43%)	41 (41%)	
	3		24 (24%)	34 (34%)	
	4		13 (13%)	6 (6%)	
	5		15 (15%)	13 (13%)	
	Missing		0 (0%)	1 (1%)	
Surgical margins					
	T2	R0	44 (84.6%)	39 (76.5%)	0.329
		R1	8 (15.4%)	12 (23.5%)	
	T3	R0	27 (56.3%)	17 (35.4%)	0.041
		R1	21 (43.8%)	31 (64.6%)	
	Overall	R0	71 (71%)	56 (56%)	0.034
		R1	29 (29%)	43 (43%)	
		Missing	0 (0%)	1 (1%)	
Patients with undetectable PSA at 3 months postoperatively (ng/mL)			85 (85%)	85 (85%)	1.000
Biochemical recurrence					0.540
	No		73 (73%)	76 (76%)	
	Yes		27 (27%)	23 (23%)	
	Missing		0 (0%)	1 (1%)	
Postoperative radiotherapy					0.605
	No		77 (77%)	78 (78%)	
	Adjuvant		1 (1%)	0 (0%)	
	Salvage		21 (21%)	21 (21%)	
	Missing		1 (1%)	1 (1%)	
Development of metastatic PCa			2 (2%)	0 (0%)	0.157
Follow-up (months)			24.5 (17–35)	22 (18–27)	0.008

ISUP: International Society of Urological Pathology; PCa: Prostate Cancer; PSA: Prostate Specific Antigen; RS-RARP: Retzius-Sparing Robot-Assisted Radical Prostatectomy; SA-RARP: Standard Anterior Robot-Assisted Radical Prostatectomy.

**Table 5:** Univariate and multivariate logistic regression analysis for biochemical recurrence in the RS-RARP group.

Variable		Odds Ratio	95% CI	*p*
**Univariate Logistic Regression**				
iPSA		1.14	1.03–1.26	0.011
pT				
	pT2			Ref
	pT3	19.06	4.15–87.53	<0.001
Lymphovascular invasion				
	No			Ref
	Yes	11.25	1.11–114.05	0.041
Surgical margin status				
	R0			Ref
	R1	3.21	1.21–8.53	0.019
ISUP Grade Group				
	1			Ref
	2–3	3.39 × 10^8^	/	1.00
	4–5	1.79 × 10^9^	/	1.00
**Multivariate Logistic Regression**				
iPSA		1.13	1.00–1.28	0.054
pT				
	pT2			Ref
	pT3	14.99	2.77–81.11	0.002
Lymphovascular invasion				
	No			Ref
	Yes	13.52	0.85–216.28	0.066
Surgical margin status				
	R0			Ref
	R1	2.05	0.56–7.54	0.281

CI: Confidence Interval; PSA: Prostate Specific Antigen.

## Data Availability

All data generated or analyzed during this study are included in this article. Further inquiries can be directed to the corresponding author.

## References

[1] Sung H., Ferlay J., Siegel R.L., Laversanne M., Soerjomataram I., Jemal A., Bray F. (2021). Global Cancer Statistics 2020: GLOBOCAN Estimates of Incidence and Mortality Worldwide for 36 Cancers in 185 Countries. CA Cancer J. Clin..

[2] Ilic D., Evans S.M., Allan C.A., Jung J.H., Murphy D., Frydenberg M. (2017). Laparoscopic and Robotic-Assisted versus Open Radical Prostatectomy for the Treatment of Localised Prostate Cancer. Cochrane Database Syst. Rev..

[3] Zahid A., Ayyan M., Farooq M., Cheema H.A., Shahid A., Naeem F., Ilyas M.A., Sohail S. (2022). Robotic Surgery in Comparison to the Open and Laparoscopic Approaches in the Field of Urology: A Systematic Review. J. Robot. Surg..

[4] Menon M., Shrivastava A., Bhandari M., Satyanarayana R., Siva S., Agarwal P.K. (2009). Vattikuti Institute Prostatectomy: Technical Modifications in 2009. Eur. Urol..

[5] Galfano A., Ascione A., Grimaldi S., Petralia G., Strada E., Bocciardi A.M. (2010). A New Anatomic Approach for Robot-Assisted Laparoscopic Prostatectomy: A Feasibility Study for Completely Intrafascial Surgery. Eur. Urol..

[6] Galfano A., Secco S., Dell’Oglio P., Rha K., Eden C., Fransis K., Sooriakumaran P., De La Muela P.S., Kowalczyk K., Miyagawa T. (2021). Retzius-Sparing Robot-Assisted Radical Prostatectomy: Early Learning Curve Experience in Three Continents. BJU Int..

[7] Asimakopoulos A.D., Topazio L., De Angelis M., Agrò E.F., Pastore A.L., Fuschi A., Annino F. (2019). Retzius-Sparing versus Standard Robot-Assisted Radical Prostatectomy: A Prospective Randomized Comparison on Immediate Continence Rates. Surg. Endosc..

[8] Umari P., Eden C., Cahill D., Rizzo M., Eden D., Sooriakumaran P. (2021). Retzius-Sparing versus Standard Robot-Assisted Radical Prostatectomy: A Comparative Prospective Study of Nearly 500 Patients. J. Urol..

[9] Menon M., Dalela D., Jamil M., Diaz M., Tallman C., Abdollah F., Sood A., Lehtola L., Miller D., Jeong W. (2018). Functional Recovery, Oncologic Outcomes and Postoperative Complications after Robot-Assisted Radical Prostatectomy: An Evidence-Based Analysis Comparing the Retzius Sparing and Standard Approaches. J. Urol..

[10] Rosenberg J.E., Jung J.H., Edgerton Z., Lee H., Lee S., Bakker C.J., Dahm P. (2021). Retzius-Sparing versus Standard Robot-Assisted Laparoscopic Prostatectomy for the Treatment of Clinically Localized Prostate Cancer. BJU Int..

[11] Lumen N., Lambert E., Poelaert F., Wirtz M., Verbeke S., Van Praet C. (2022). Retzius-Sparing Robot-Assisted Radical Prostatectomy. J. Vis. Exp..

[12] Bianchi L., Turri F.M., Larcher A., De Groote R., De Bruyne P., De Coninck V., Goossens M., D’Hondt F., De Naeyer G., Schatteman P. (2018). A Novel Approach for Apical Dissection During Robot-Assisted Radical Prostatectomy: The “Collar” Technique. Eur. Urol. Focus.

[13] Clavien P.A., Barkun J., de Oliveira M.L., Vauthey J.N., Dindo D., Schulick R.D., de Santibañes E., Pekolj J., Slankamenac K., Bassi C. (2009). The Clavien-Dindo Classification of Surgical Complications. Ann. Surg..

[14] Barakat B., Othman H., Gauger U., Wolff I., Hadaschik B., Rehme C. (2022). Retzius Sparing Radical Prostatectomy Versus Robot-Assisted Radical Prostatectomy: Which Technique Is More Beneficial for Prostate Cancer Patients (MASTER Study)? A Systematic Review and Meta-Analysis. Eur. Urol. Focus.

[15] Galfano A., Di Trapani D., Sozzi F., Strada E., Petralia G., Bramerio M., Ascione A., Gambacorta M., Bocciardi A.M. (2013). Beyond the Learning Curve of the Retzius-Sparing Approach for Robot-Assisted Laparoscopic Radical Prostatectomy: Oncologic and Functional Results of the First 200 Patients with ≥1 Year of Follow-Up. Eur. Urol..

[16] Umari P., Fossati N., Gandaglia G., Pokorny M., De Groote R., Geurts N., Goossens M., Schatterman P., De Naeyer G., Mottrie A. (2017). Robotic Assisted Simple Prostatectomy versus Holmium Laser Enucleation of the Prostate for Lower Urinary Tract Symptoms in Patients with Large Volume Prostate: A Comparative Analysis from a High Volume Center. J. Urol..

[17] Qiu X., Li Y., Chen M., Xu L., Guo S., Marra G., Elliot Rosenberg J., Ma H., Li X., Guo H. (2020). Retzius-Sparing Robot-Assisted Radical Prostatectomy Improves Early Recovery of Urinary Continence: A Randomized, Controlled, Single-Blind Trial with a 1-Year Follow-Up. BJU Int..

[18] Lim S.K., Kim K.H., Shin T.Y., Han W.K., Chung B.H., Hong S.J., Choi Y.D., Rha K.H. (2014). Retzius-Sparing Robot-Assisted Laparoscopic Radical Prostatectomy: Combining the Best of Retropubic and Perineal Approaches. BJU Int..

[19] Dalela D., Jeong W., Prasad M.A., Sood A., Abdollah F., Diaz M., Karabon P., Sammon J., Jamil M., Baize B. (2017). A Pragmatic Randomized Controlled Trial Examining the Impact of the Retzius-Sparing Approach on Early Urinary Continence Recovery After Robot-Assisted Radical Prostatectomy. Eur. Urol..

[20] Sayyid R.K., Simpson W.G., Lu C., Terris M.K., Klaassen Z., Madi R. (2017). Retzius-Sparing Robotic-Assisted Laparoscopic Radical Prostatectomy: A Safe Surgical Technique with Superior Continence Outcomes. J. Endourol..

[21] Egan J., Marhamati S., Carvalho F.L.F., Davis M., O’Neill J., Lee H., Lynch J.H., Hankins R.A., Hu J.C., Kowalczyk K.J. (2021). Retzius-Sparing Robot-Assisted Radical Prostatectomy Leads to Durable Improvement in Urinary Function and Quality of Life Versus Standard Robot-Assisted Radical Prostatectomy Without Compromise on Oncologic Efficacy: Single-Surgeon Series and Step-by-Step. Eur. Urol..

[22] Stonier T., Simson N., Davis J., Challacombe B. (2019). Retzius-Sparing Robot-Assisted Radical Prostatectomy (RS-RARP) vs Standard RARP: It’s Time for Critical Appraisal. BJU Int..

[23] Chang L.-W., Hung S.-C., Hu J.-C., Chiu K.-Y. (2018). Retzius-Sparing Robotic-Assisted Radical Prostatectomy Associated with Less Bladder Neck Descent and Better Early Continence Outcome. Anticancer Res..

[24] Abdel Raheem A., Hagras A., Ghaith A., Alenzi M.J., Elghiaty A., Gameel T., Alowidah I., Ham W.S., Choi Y.D., El-Bahnasy A.H. (2020). Retzius-Sparing Robot-Assisted Radical Prostatectomy versus Open Retropubic Radical Prostatectomy: A Prospective Comparative Study with 19-Month Follow-Up. Minerva Urol. Nefrol..

[25] Nyberg M., Sjoberg D.D., Carlsson S.V., Wilderäng U., Carlsson S., Stranne J., Wiklund P., Steineck G., Haglind E., Hugosson J. (2021). Surgeon Heterogeneity Significantly Affects Functional and Oncological Outcomes after Radical Prostatectomy in the Swedish LAPPRO Trial. BJU Int..

[26] Lantz A., Bock D., Akre O., Angenete E., Bjartell A., Carlsson S., Modig K.K., Nyberg M., Kollberg K.S., Steineck G. (2021). Functional and Oncological Outcomes After Open Versus Robot-Assisted Laparoscopic Radical Prostatectomy for Localised Prostate Cancer: 8-Year Follow-Up. Eur. Urol..

[27] Phukan C., Mclean A., Nambiar A., Mukherjee A., Somani B., Krishnamoorthy R., Sridhar A., Rajan P., Sooriakumaran P., Rai B.P. (2020). Retzius Sparing Robotic Assisted Radical Prostatectomy vs. Conventional Robotic Assisted Radical Prostatectomy: A Systematic Review and Meta-Analysis. World J. Urol..

[28] Checcucci E., Veccia A., Fiori C., Amparore D., Manfredi M., Di Dio M., Morra I., Galfano A., Autorino R., Bocciardi A.M. (2020). Retzius-Sparing Robot-Assisted Radical Prostatectomy vs the Standard Approach: A Systematic Review and Analysis of Comparative Outcomes. BJU Int..

[29] Tai T.E., Wu C.C., Kang Y.N., Wu J.C. (2020). Effects of Retzius Sparing on Robot-Assisted Laparoscopic Prostatectomy: A Systematic Review with Meta-Analysis. Surg. Endosc..

[30] Abdel Raheem A., Chang K.D., Alenzi M.J., Ham W.S., Han W.K., Choi Y.D., Rha K.H. (2018). Predictors of Biochemical Recurrence after Retzius-Sparing Robot-Assisted Radical Prostatectomy: Analysis of 359 Cases with a Median Follow-up Period of 26 Months. Int. J. Urol..

[31] Menon M., Bhandari M., Gupta N., Lane Z., Peabody J.O., Rogers C.G., Sammon J., Siddiqui S.A., Diaz M. (2010). Biochemical Recurrence Following Robot-Assisted Radical Prostatectomy: Analysis of 1384 Patients with a Median 5-Year Follow-Up. Eur. Urol..

[32] Galfano A., Tappero S., Eden C., Dell’Oglio P., Fransis K., Guo H., Kowalczyk K., Longoni M., Madi R., Rha K.H. (2022). Multicentric Experience in Retzius-Sparing Robot-Assisted Radical Prostatectomy Performed by Expert Surgeons for High-Risk Prostate Cancer. Minerva Urol. Nephrol..

[33] Lee J., Kim H.Y., Goh H.J., Heo J.E., Almujalhem A., Alqahtani A.A., Chung D.Y., Chang K., Choi Y.D., Rha K.H. (2020). Retzius Sparing Robot-Assisted Radical Prostatectomy Conveys Early Regain of Continence over Conventional Robot-Assisted Radical Prostatectomy: A Propensity Score Matched Analysis of 1,863 Patients. J. Urol..

[34] Anil H., Karamik K., Yildiz A., Savaş M. (2021). Does Transition from Standard to Retzius-Sparing Technique in Robot-Assisted Radical Prostatectomy Affect the Functional and Oncological Outcomes?. Arch. Ital. Urol. Androl..

[35] Kates M., Sopko N.A., Han M., Partin A.W., Epstein J.I. (2016). Importance of Reporting the Gleason Score at the Positive Surgical Margin Site: Analysis of 4,082 Consecutive Radical Prostatectomy Cases. J. Urol..

